# Projection of the Climate-Suitable Area of the Invasive Pest *Phoracantha semipunctata* (Coleoptera: Cerambycidae: Phoracantha) and Its Ability to Continue to Expand in China

**DOI:** 10.3390/insects16111171

**Published:** 2025-11-17

**Authors:** Kaitong Xiao, Ruixiong Deng, Xin Chen, Ciai Yu, Lin Wu, Hang Ning, Hui Chen

**Affiliations:** 1 Hubei Key Laboratory of Biological Resources Protection and Utilization, Hubei Minzu University, Enshi 445000, China; 13402709191@163.com (K.X.);; 2 College of Forestry and Horticulture, Hubei Minzu University, Enshi 445000, China; 3 State Key Laboratory for Conservation and Utilization of Subtropical Agro-Bioresources, Guangdong Key Laboratory for Innovative Development and Utilization of Forest Plant Germplasm, College of Forestry and Landscape Architecture, South China Agricultural University, Guangzhou 510642, China

**Keywords:** *Phoracantha semipunctata*, climate change, Random Forests, potential geographical distribution, pest management

## Abstract

*Phoracantha semipunctata* is a global quarantine pest, which is fatal to various tree species of the *Eucalyptus*. Currently, this pest has landed and colonized Guangdong province, China. Previously, there was very limited research information on *P. semipunctata* in China, which basically describes the taxonomic status. Field investigations found that the climatic ecological niche of the pest is continuing to expand. With global warming and the globalization of trade, signs of expansion may intensify the spread. In order to prevent any further spread of *P. semipunctata*, it is important to clarify its geographic distribution in China. In this study, the algorithm Random Forests was used to project the potential geographic distribution of *P. semipunctata* in China currently and in the future. Our results showed that temperature seasonality (Bio4) and the precipitation of the coldest quarter (Bio19) are key environmental factors limiting the current distribution of *P. semipunctata* in China. Currently, *P. semipunctata* has been found in Guangdong province, colonizing in the triangle zone composed of Zhanjiang City, Qingyuan City, and Jieyang City, with the projected potential suitable distribution area of 50.88 × 10^4^ km^2^. Under future climate scenarios, the total suitable distribution area is projected to increase, from Fujian province toward the north to Guangxi province toward the north. Building on these results, we predicted the potential future spread pattern of *P. semipunctata* and developed priority measures for its management. These findings provide a theoretical basis for designing effective quarantine and control strategies against *P. semipunctata*.

## Simple Summary

A harmful beetle (*Phoracantha semipunctata*) that kills eucalyptus has invaded China and is now established in Guangdong province. This invasive beetle species is likely to spread further because of climate change and increased global trade, but it was not clear where it could survive across the country. To address this threat, we used a computer model to identify the key factors affecting its distribution and predict areas suitable for this beetle now and in the future, in China, based on climate change conditions, for the first time. The result shows that this beetle’s survival is mainly limited by temperatures varying between seasons and rainfall during the coldest quarter. Currently, large areas of southern China (over 500,000 km^2^), especially in Guangdong, have suitable conditions for it. As the climate warms, this suitable area is expected to grow, spreading northwards, particularly into Fujian and Guangxi provinces. Furthermore, the prioritized measures for pest control strategies were formulated. Therefore, our findings provide a theoretical basis for implementing quarantine and control strategies aimed at *P. semipunctata.*

## 1. Introduction

*Eucalyptus* L’Hér. plays a vital role in global carbon sequestration and paper production [1]. It has been in China for more than 120 years, gradually developing into an important tree species for fast-growing, productive forests in the south of China. Guangdong province and Guangxi province have become a prominent part of China’s timber and forestry production industries. The warm, humid climate and fertile soil conditions provide a favorable environment for the growth of *eucalyptus*, promoting a rapid increase in the areas planted in these provinces [2]. At present, the process of planting eucalyptus and its scientific management is mainly based on rough operations. This resulted in the abnormal growth of the forest stand, causing its growth to weaken from year to year [1]. In addition, eucalyptus plantations created in a short period of time are often of one species or even of the same age of the same variety [3]. Therefore, once the hydrothermal conditions are suitable, the pests and diseases are beneficial for spreading rapidly, proliferation, epidemic, large-scale outbreaks, leading to the morbidity or death of patches of forests and trees, causing the inestimable economic and ecological losses, seriously restricting the development of the local economy [4,5].

*Phoracantha semipunctata* Fabricius, a eucalyptus long-horned borer, is native to Australia and is a destructive eucalyptus forest pest [6,7]. Currently, the pest has spread to all continents except Antarctica via the trade transportation of wood and wood packaging materials [8]. *P. semipunctata* feeds on eucalyptus, with the adult damaging almost all parts of trees and the larvae developing in almost all major tissues of the hosts [9]. In Australia, *P. semipunctata* is primarily a killer of dead and felled trees, while, in all the other countries where this pest has invaded, it is extremely damaging to dead wood and living trees [10]. In China, *P. semipunctata* was included in the list of imported plant quarantine pests. In recent years, the Chinese port inspection and quarantine bureaus have intercepted *P. semipunctata* on several occasions [6]. In the face of an expansion of invasive populations, the large number of eucalyptus trees artificially planted in the southeastern provinces of China will inevitably suffer severe threats. Based on this, integrated prevention and control of *P. semipunctata* has already been placed on the agenda. However, traditional physical and chemical control measures are unable to effectively treat *P. semipunctata* due to the lack of detailed invasion history information about this beetle. Consequently, confronting the current crisis, the adverse situations are expected to become increasingly serious.

The global warming trend is continuing. In 2019, the global average temperature was about 1.1 °C above the pre-industrial level, and the average temperature of the land surface in Asia was 0.87 °C higher than the normal value (the report is based on the climatic base period of 1981–2010) [11]. China is a sensitive area and an area that has a significant impact on global climate change [12]. From 1951 to 2019, the annual average temperature in China increased by 0.24 °C per decade, with a rate of warming significantly higher than that of the global average over the same period; the past 20 years have been the warmest period since the beginning of the 20th century [13]. Temperature is an important environmental factor that determines the geographical distribution and growth and development of insects [14]. Climate warming, marked by rising temperatures, will inevitably have a significant impact on the geographical distribution and growth and development of insects [15]. Numerous studies have found that climate warming has widened the habitat range of insects, accelerating their growth and development rate, leading to the continuous expansion of the geographical distribution of insects [16]. Field Investigations of the populations found that the geographical range of *P. semipunctata* has expanded. New occurrence records are being discovered all the time. Continuous climate change is likely to change the life-history characteristics of this pest, causing shifts in species’ geographical distributions on an ongoing basis. It is therefore critical to figure out how its geographical distribution in China will respond to climate change, to enable appropriate ongoing pest prevention and control management. To address this, the Random Forest algorithm was applied to project climate-change-driven potential distribution alterations for this pest.

Random Forest (RF), an ensemble machine learning method, is being broadly used to project the potential distribution of target species [17]. RF predicts responses by averaging multiple regression trees, each using a random subset of all model variables, and it deals with highly covariant predictors by propagating variable importance across all variables [18,19]. Hence, RF can effectively avoid the shortcomings associated with over-fitting of previous species distribution models (SDMs), thereby obtaining more accurate and stable predictions. At the same time, the RF algorithm efficiently handles large datasets and performs robustly even with missing values [17,20]. Owing to its strong performance and wide applicability, numerous studies have demonstrated the accuracy and reliability of RF in modeling species distributions [21,22]. Therefore, RF was employed to estimate the potential geographic range of the target species under different climate change scenarios.

Pests of *Eucalyptus* showed a trend towards severe incidence, which, to some extent, is closely linked to climate change. Particularly, warm winters and extreme climate anomalies favor the overwintering of pests, providing the conditions for major occurrences the following year [23]. Additionally, successive droughts and high temperatures also caused host *Eucalyptus* stands to decline and even die, which leads to increased stand susceptibility, thereby raising the frequency of pest outbreaks [24]. Consequently, one of the critical questions is: how should the *P. semipunctata* population, which has already been colonized in China, respond to climate change? To answer this question, RF was used in this study to predict climate change-driven potential distribution alterations, which may be the key to understanding the fates of this population in China. This research has three objectives: (1) to identify the dominant environmental variables that limit the distribution range of the *P. semipunctata* population; (2) to project distributions of *P. semipunctata* in China currently and under climate change scenarios in the future; (3) to provide prevention and control management strategies for the *P. semipunctata* population based on these findings.

## 2. Materials and Methods

### 2.1. Presence Records of P. semipunctata

**Figure 1:**
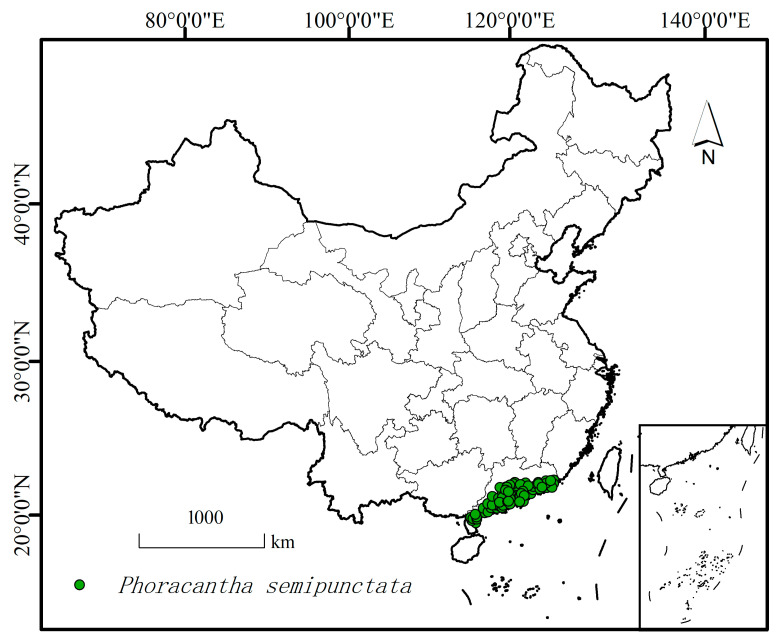
Current occurrence records of *P. semipunctata* in China.

We gathered the presence data of *P. semipunctata* through four methods. First, we collected occurrence records of *P. semipunctata* from 2022 to 2024 via field investigations with the assistance of the Department of Forestry Protection. The categories of location name, longitude, and altitude were documented for all occurrence points. The distance between the investigated points was greater than 20 km. Second, we searched the Chinese Animal and Plant Quarantine Service Information Resources Sharing Platform (General Administration of Customs of the People’s Republic of China, http://gdfs.customs.gov.cn/, accessed on 15 October 2023) to find data concerning interceptions of *P. semipunctata*. This database included information on the port where the interception was made, the dates of the interceptions, and the number of specimens found. Third, we downloaded and arranged presence data from the websites of the Global Biodiversity Information (GBIF, https://doi.org/10.15468/dl.5npp9e, accessed on 29 November 2023) [25]. We searched the web using its scientific name to find information about the worldwide distribution, damage, and colonization. Coordinates with complete latitude and longitude will be chosen directly, while the remaining incomplete locations where only names were provided will obtain the latitude and longitude by using Google Maps. In China, we obtained three distinct occurrence locations for this pest from the GBIF website [25]. Among them, two points were located in Guangzhou City, Guangdong province, which were identified as *P. semipunctata* presence points after comparison with the actual locations. However, the remaining point from Yunnan province was not found to be present after the actual survey. This one point was therefore discarded. Fourth, relevant presence records were collected from the literature, while occurrence data with incomplete geographic information and minor differences in geographic coordinates were deleted. Therefore, a total of 52 presence points were preliminarily collected. To reduce sampling bias caused by overfitting and imprecise spatial records, we applied the “Spatial Rarity Occurrence Data” tool in the SDM Toolbox of ArcGIS 10.4 (Environmental Systems Research Institute, Redlands, CA, USA) to refine the occurrence data, retaining only one record per 10 × 10 km^2^ grid [26]. In total, 45 filtered occurrence points were used as the presence data for *P. semipunctata* in the subsequent potential distribution modeling with RF (Figure 1).

A bioclimatic profile of *P. semipunctata* was obtained from descriptive statistical analysis via Excel 2016 (Microsoft Corp., Redmond, WA, USA). The profile characterizes the environmental conditions of habitats where *P. semipunctata* occurs in China and serves to identify other areas potentially suitable for its establishment. The mean, standard deviation, and range of environmental variables were calculated to define the species’ suitability thresholds.

### 2.2. Environmental Data

In order to explore the environmental variables affecting the distribution of *P. semipunctata*, 19 bioclimatic variables and three topographic variables were selected to participate in the modeling (Table 1). A total of 19 bioclimatic layers with 2.5′ resolution were obtained from the WorldClim online database (http://www.worldclim.org/, accessed on 25 October 2023). The altitude layer with a spatial resolution of 30 m was derived from the digital elevation model (DEM) of the geospatial data cloud online database (http://www.gscloud.cn/, accessed on 25 October 2023). To be consistent with the resolution of the bioclimatic variables, the resolution of the DEM was converted to 2.5′ via using the resample tool with the Nearest Neighbor algorithm in ArcGIS 10.4. Slope and aspect layers were calculated based on the altitude layer using the slope and aspect tools in ArcGIS 10.4, respectively. The analytical base map was downloaded from the National Fundamental Geographic Information System (http://www.ngcc.cn, accessed on 25 October 2023).

Future climate scenarios are obtained from the WorldClim, which uses the thin plate smoothing spline function interpolation method to obtain the current climate layer according to the observed data of meteorological stations all over the world [27]. The period of the future climate layer includes the 2050s (2041–2060) and the 2070s (2061–2080). Global climate model BCC-CSM1.1 was chosen as the climate model for modelling in this study. A total of four representative concentration pathways (RCPs) were announced in the fifth Intergovernmental Panel on Climate Change report (IPCC AR5) [11]. The total radiative forcing in 2100 in these four RCPs had reached 2.6 W/m^2^ (RCP2.6), 4.5 W/m^2^ (RCP405), 6 W/m^2^ (RCP6.0), and 8.5 W/m^2^ (RCP8.5) over the values in 1750 [28]. In this study, RCP2.6, RCP4.5, RCP6.0, and RCP8.5 were selected to project the future distributions of *P. semipunctata* in the 2050s and 2070s.

The Pearson correlation coefficients (r) and the least absolute shrinkage and selection (LASSO) were used sequentially to filter for important environmental factors affecting the potential distribution of targeted species. Certainly, this dimensionality reduction process is also aimed at eliminating the over-fitting of the models due to multicollinearity between these environmental factors, thereby improving the accuracy of the model. First, the Pearson correlation coefficients (r) were used to calculate the correlation between each of the two environmental variables out of the 22 variables. Once there is a correlation between two variables greater than 0.8, only one of them is retained [29]. Next, the LASSO algorithm is used to construct a penalty function that compresses the coefficients of some variables and shrinks some of them to 0. We retain all environmental variables with non-zero coefficients as the final key variables [30,31].

### 2.3. P. semipunctata Distribution Predicted by RF

Firstly, 180 pseudo-presence coordinates using ArcGIS 10.4 were randomly generated to avoid sample bias caused by the occurrence records from different sources in the study area. All the presence and pseudo-presence coordinates were merged into a single coordinate dataset to as a point-based input dataset. Then, all the environment variables and the input dataset are loaded together for modeling using an RF model, which can be realized by the BioMod2 package in R. We used 10-fold cross-validation to train and validate the model. Of the 10 subsets, a single subset was retained as the validation data for testing the model, and the remaining subsets were used as training data. For each subset, 70% of the input dataset was used to train the single model, and the remaining dataset was used to test the predictive performance of the model. After the model was run 10 times with cross-validation, the optimal model was selected for subsequent generalization calculations. Additionally, we used the corresponding statistical analysis to obtain the contribution of each environmental variable to the model predictions for each climate scenario.

AUC, TSS, and Kappa values were used to evaluate the accuracy of the model under each climate scenario (Table 2). AUC is the area under the receiver operating characteristic curve (ROC), with values ranging from 0 to 1 [32]. The Kappa coefficient is an index to evaluate the classification accuracy of the model based on the confusion matrix, with a range of −1 to 1. True skill statistics (TSS) is an improved test index derived from the kappa coefficient, which was used to evaluate the discrimination ability of the model via calculating the difference between the true positive rate and the false positive rate when the model predicts the positive and negative classes, with values ranging from 0 to 1 [33]. The closer the value of the above three indices is to 1, the higher the accuracy of the model. The detailed evaluation indicators for model accuracy are shown in Table 2.

We used the habitat suitability index (HSI), which has been widely used to evaluate habitat suitability, to classify the suitable distribution of *P. semipunctata* in this study [34]. Eventually, the habitat suitability of *P. semipunctata* in the study area was classified into four categories: unsuitable (0–0.3), marginally suitable (0.31–0.5), moderately suitable (0.51–0.7), and highly suitable (0.71–1). Subsequently, we calculated the area of each suitable distribution for *P. semipunctata* under different climate scenarios, though the number of presence grid cells multiplied by their spatial resolution.

## 3. Results

### 3.1. Bioclimatic Profile of P. semipunctata

By conducting statistical analysis on the climate data for each *P. semipunctata* distribution coordinate, we obtained the bioclimatic profile of *P. semipunctata,* which suggests the temperature and precipitation adaptation range of this pest (Table 3).

### 3.2. Filtering of Important Environmental Variables and Model Performance

**Figure 2:**
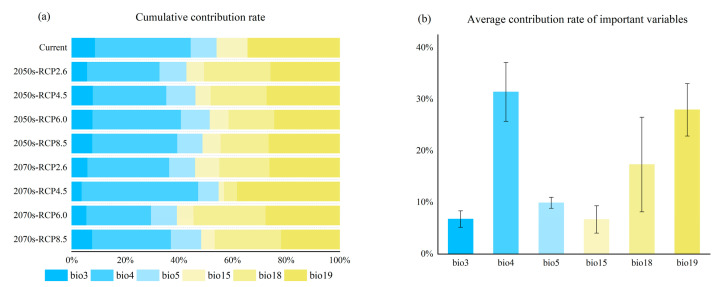
Sorting and filtering of important variables affecting the distribution of *P. semipunctata*: (**a**) Contribution rates of important variables under different climate scenarios; (**b**) Average contribution rates of important variables (Bio3: Isothermality, Bio4: temperature seasonality, Bio5: max temperature of warmest month, Bio15: Precipitation seasonality, Bio18: precipitation seasonality and Bio19: precipitation coldest quarter).

First, the contribution weight of each environmental variable influencing the distribution of *P. semipunctata* was calculated, and those variables accounting for a cumulative contribution of 100% under each climate scenario were selected. Then, we calculated the average contribution of the above variables. The results of random forest modeling showed that a total of six variables maintain robust contribution rates in different climate scenarios (Figure 2). Six key environmental variables were identified in descending order of contribution: Bio4 (temperature seasonality), Bio19 (precipitation of coldest quarter), Bio18 (precipitation seasonality), Bio5 (maximum temperature of warmest month), Bio3 (isothermality), and Bio15 (precipitation seasonality). Moreover, the cumulative contribution of temperature-related factors (blue columns) exceeded that of precipitation-related ones (yellow columns), indicating that *P. semipunctata* is more sensitive to temperature variation than to rainfall. This finding enhances our understanding of the beetle’s potential responses to climate change.

**Figure 3:**
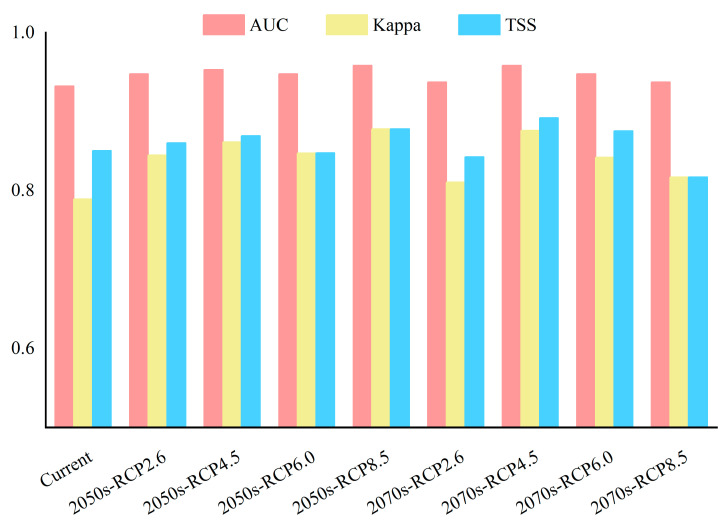
Evaluation indicators for the prediction accuracy of *P. semipunctata* in current and future climate scenarios. (AUC > 0.95, Kappa > 0.8, and TSS > 0.85, suggesting high accuracy of the model).

Figure 3 shows the values of the indices AUC, Kappa, and TSS for assessing the accuracy of modeling under different climate scenarios. Under all climate scenarios, the results of modeling show that the AUC value is greater than 0.95, the Kappa value is greater than 0.8, and the TSS value is greater than 0.85, suggesting there is a high accuracy of the results predicted by the RF model.

### 3.3. Potential Distribution of P. semipunctata in China from the Current to the Future

#### 3.3.1. Potential Distribution of *P. semipunctata* in the Current Climate Scenario

**Figure 4:**
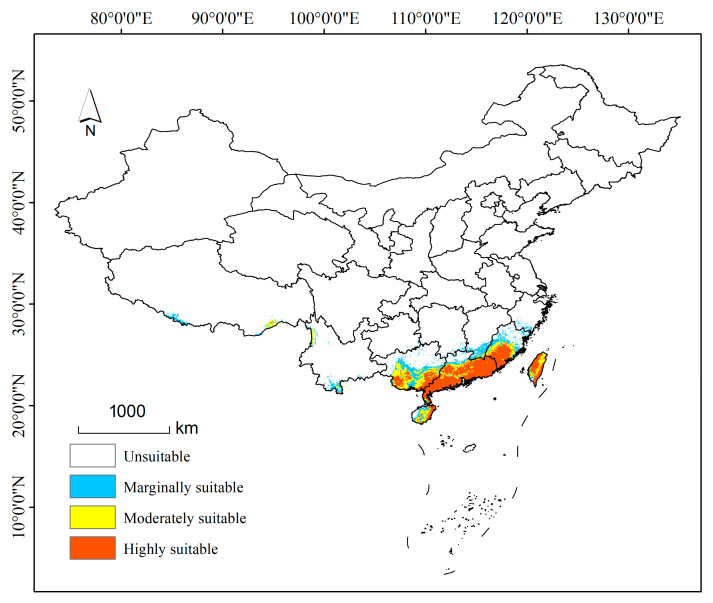
Potential distribution of *P. semipunctata* in China under the current climate scenario. (unsuitable: 0–0.3, marginally suitable: 0.31–0.5, moderately suitable: 0.51–0.7, and highly suitable: 0.71–1).

**Figure 5:**
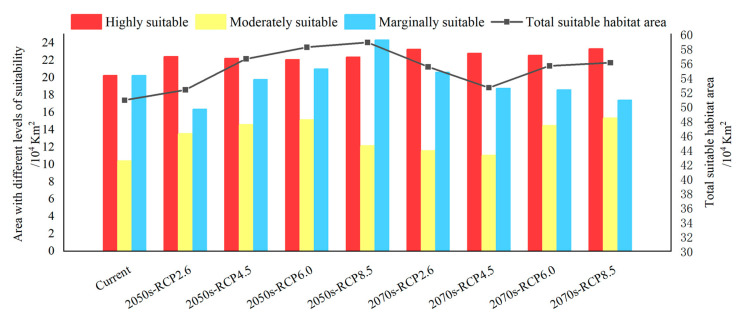
Suitable area by suitability class of *P. semipunctata* under different climate scenarios.

The current distribution of *P. semipunctata* in China is shown in Figure 4. *P. semipunctata* is mainly distributed in Guangdong Province, Guangxi Province, Fujian Province, Taiwan Province, and Hainan Province. Additionally, there are a small number of potential distribution areas distributed in Yunnan Province, Tibet Province, Guizhou Province, Jiangsu Province, Jiangxi Province, and Hunan Province. Currently, the total suitable area of *P. semipunctata* amounts to 50.88 × 10^4^ km^2^, among which the highly suitable area, moderately suitable area, and marginally suitable area are approximately 20.24 × 10^4^ km^2^, 10.4 × 10^4^ km^2^, and 20.24 × 10^4^ km^2^, respectively (Figure 5).

#### 3.3.2. Potential Distribution of *P. semipunctata* Under Future Climate Scenarios

**Figure 6:**
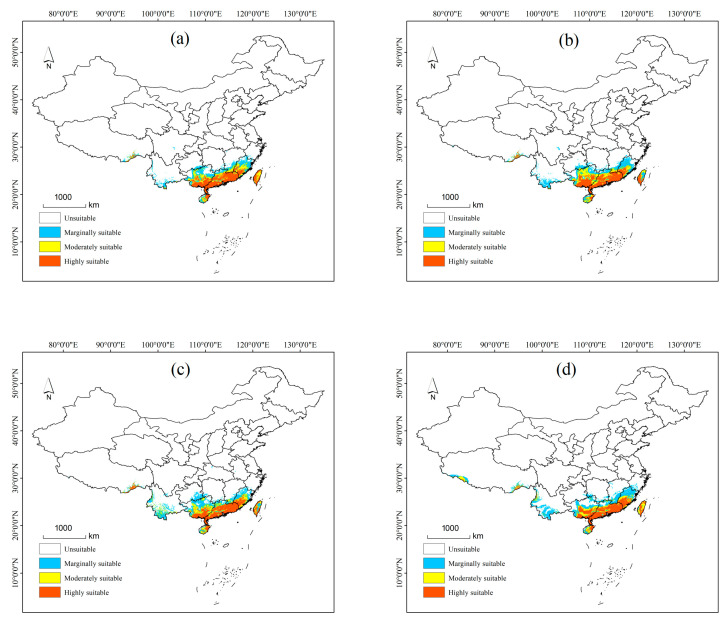
Potential distribution of *P. semipunctata* in China in 2050s: (**a**) in the RCP2.6 scenario; (**b**) in the RCP4.5 scenario; (**c**) in the RCP6.0 scenario; (**d**) in the RCP8.5 scenario. (unsuitable: 0–0.3, marginally suitable: 0.31–0.5, moderately suitable: 0.51–0.7 and highly suitable: 0.71–1).

**Figure 7:**
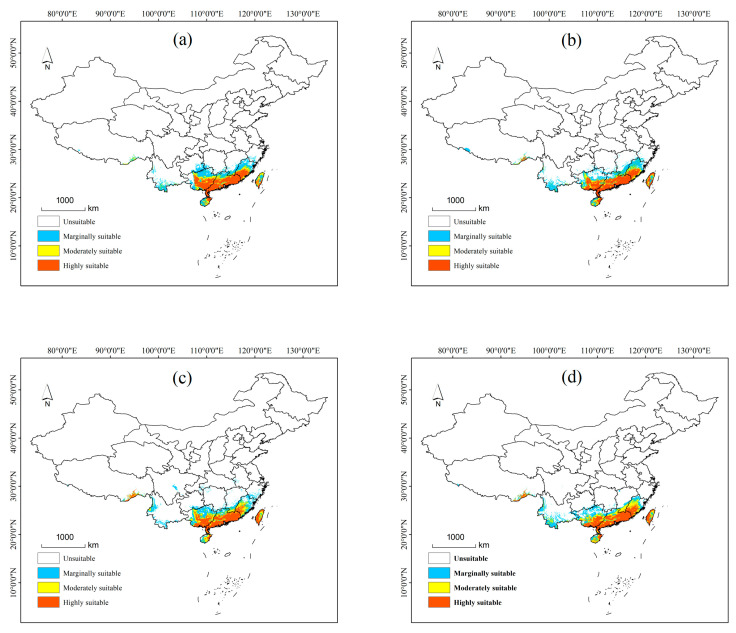
Potential distribution of *P. semipunctata* in China in 2070s: (**a**) in the RCP2.6 scenario; (**b**) in the RCP4.5 scenario; (**c**) in the RCP6.0 scenario; (**d**) in the RCP8.5 scenario. (unsuitable: 0–0.3, marginally suitable: 0.31–0.5, moderately suitable: 0.51–0.7 and highly suitable: 0.71–1).

Under future climate scenarios, the potential distribution of *P. semipunctata* is shown in Figure 6 and Figure 7. Under most future climate scenarios, the total suitable distribution range showed an increasing trend, coming mainly from Fujian Province toward the north, Guangxi Province toward the north, and Yunnan Province toward the southwest. Among them, the highly and moderately suitable area is projected to increase somewhat in the near future; the marginally suitable distribution under most climate scenarios is projected to decline somewhat.

Under the RCP2.6-2050s climate scenario, the total suitable area has increased by 1.44 × 10^4^ km^2^. Compared with the current distribution, the highly and moderately suitable areas increased by 10.85% and 30.12%, respectively, while the marginally suitable distribution decreased by 19.17%. In the 2070s, the highly, moderately, and marginally suitable areas increased by 15.02%, 11.29%, and 1.97%, respectively, with the total suitable area amounting to 55.49 × 10^4^ km^2^. The largest alterations in response to RCP 2.6 climate scenarios occurred in northern Guangxi Province.

Under the RCP4.5-2050s climate scenario, the total suitable area has increased by 5.73 × 10^4^ km^2^. Of these, the moderately suitable distribution increased by 40.35%, while the marginally suitable area decreased by 2.26%. In the 2070s, the total suitable area has been reduced by 4 × 10^4^ km^2^ than in the 2050s, where it is mainly a moderately suitable distribution in Guangxi Province and Guangdong Province.

Under the RCP6.0-2050s climate scenario, the total suitable area has increased by 7.32 × 10^4^ km^2^. Among them, the highly, moderately, and marginally suitable areas have increased by 9.03%, 45.49%, and 3.83%, respectively. In the 2070s, the moderately suitable area has increased by 4.07 × 104 km^2^ more than the current distribution, and this has mainly come from southern Yunnan province; while the marginally suitable area has been reduced by 1.64 × 10^4^ km^2^, mainly showing in southeastern Guizhou province.

Under the RCP6.0-2050s climate scenario, the total suitable area has increased by 7.98 × 10^4^ km^2^. Of these, the highly, moderately, and marginally suitable areas increased by 10.46%, 16.91%, and 20.33%, respectively. The marginally suitable area in southwest Yunnan province and southern Zhejiang province showed a significant increase. In the 2070s, the highly and moderately suitable areas increased by 15.20% and 47.56%, respectively, and this is mainly from northern Fujian Province and northern Guangxi Province.

## 4. Discussion

According to the China Forestry Development Report, annual outbreaks of forest pests and diseases in China can cause the destruction of about 8.6 million hectares of forest resources, a huge economic loss of more than CNY 88 billion, with invasive pests being one of the main causes of the destruction of forest resources [35,36]. The southeastern coastal provinces, as the front line of invasive pests landing, China Entry-Exit Inspection and Quarantine Bureau intercepts more and more batches of quarantine pests every year, and the types of intercepted species are more and more complex and diversified [37,38]. Previously, some reports or studies on *P. semipunctata* have repeatedly shown that the pest has only been recorded in several interceptions, with no reports of survival in China [7,8]. However, field surveys over the past three years have revealed that the pest has colonized some cities in Guangdong province. To prevent the further spread of this pest within China, our projected analysis makes it possible to classify and monitor its habitat in a targeted way, based on habitat suitability maps. These results not only formulate effective measures of integrated control but also improve the efficiency of monitoring work.

The projected current potential distribution of *P. semipunctata* showed that there are large suitable areas for survival in Southern China, mainly including Guangdong province, Guangxi province, Fujian Province, Taiwan Province, Hainan Province, and some marginally suitable areas in other provinces. The total suitable distribution area showed an increasing trend in the future. The future projections maps showed future increases in suitable distribution come mainly from Fujian Province toward the north and Guangxi Province toward the north. It is worth noting that *P. semipunctata* has already colonized cities such as Zhanjiang, Qingyuan, Guangzhou, Jieyang, and Shenzhen. These cities are important and networked locations for trade with neighboring provinces, with a large radiation area, which greatly increases the probability of the pest spreading outward by timber and seedlings transportation. Currently, the colonization position of *P. semipunctata* in Guangdong map forms a triangle zone composed of Zhanjiang City, Qingyuan City, and Jieyang City, which provides climatic and geographical prerequisites for the spread of the pest. Zhanjiang City is located on the Leizhou Peninsula, which is geographically well-connected to Hainan Island to the south and to Guangxi Province to the north. Jieyang City is located in eastern Guangdong Province, which is an important transportation hub connecting Fujian Province and Jiangxi Province. Qingyuan City is located in the north-central Guangdong Province, which is connected to Hunan Province and Guangxi Province in the north. Therefore, the triangle zone provides a convenient and necessary route for the spread of the pest to the northern or southwestern regions, which is highly consistent with the results of our projections. Furthermore, we prioritized measures for pest control strategies based on these results.

In this study, the variable screening results indicated that temperature seasonality (bio4) and the precipitation of the coldest quarter (bio19) were the primary factors constraining the current distribution of *P. semipunctata* in China. Based on the global invasion history, we found *P. semipunctata* mostly colonized in the Mediterranean and coastal ranges worldwide, in which rich light, heat, and precipitation resources enable this pest to survive under many climatic constraints. More and more research has found that temperature and precipitation patterns play a crucial role in the colonization process and geographic distribution of a species [39]. The spawning period of *P. semipunctata* is from March to September each year. The pupal period, which starts from early spring to late summer, lasts for 32–40 days, while, in autumn, it lasts for approximately 110 days. When adults begin to emerge in spring or midsummer, they usually complete within 2–3 months, while they can emerge for up to 9 months from late summer [40]. Considering 11.5 °C as the minimum threshold temperature, Gonzalez Tirado estimated the thermic integral needed to complete one generation as 1510 degree-days [41]. One, two, or three generations per year may occur in Mediterranean climates, while only a single generation occurs in the California climate. These findings greatly support the idea that *P. semipunctata* prefers to survive in warm climatic conditions, which allows more generations of *P. semipunctata* per year than in other climates. Observations of field populations in Guangdong province suggest that temperatures below −15 °C reduced larval survival. Consequently, the above-mentioned findings suggested that the climate environmental conditions of the eastern coastal provinces of China and the islands of Hainan and Taiwan range are favorable for *P. semipunctata* survival. In addition, for many invasive species, synchronous adult emergence and life-cycle timing are required to kill the host trees [42]. *P. semipunctata* has an obligatory winter diapause period, which appears to synchronize larval and pupal development [43]. Whether larval populations were initiated in May, June, or October, the resulting adults emerged the following year between early June and early August [44]. Synchronous adult emergence is an important regulator of insect seasonality and synchrony, and ultimately of the mean fitness of the population. To improve their chances of surviving adverse conditions, such as extreme cold or heat, the vulnerable stages of the life cycle need to coincide with favorable seasonal conditions, a phenomenon referred to as seasonality [42,45]. Consequently, suitable seasonality is essential for the population growth and outbreak potential of *P. semipunctata*.

The global colonization history of *P. semipunctata* shows that this pest is present in almost all countries or regions where Eucalyptus species have been introduced [7,46]. In this study, host distribution as a predictor variable has little weight for the potential distribution of the pest. Currently, hosts widely planted in southern China are distributed far more widely than *P. semipunctata*. Therefore, what we are more concerned about is that the potential distribution of the pest is limited more by climate variables at the macro-scale level. The field survey found that *P. semipunctata,* which has been colonized in Guangdong, China, has established stable climate-related populations. Consequently, we need to give more thought to the limitations where the populations might spread. Generally, invasive species arriving in zones with environmental conditions similar to those of their native or colonized zones are more likely to successfully establish populations. Undoubtedly, China possesses a more complete and diverse range of climatic types compared to the native habitats and colonized areas of this pest. Therefore, without considering the constraints of the geographic distribution of hosts, northern China may be a viable area for *P. semipunctata*. The spread of this pest along the coastal provinces of China may be an important route, which would be the first choice for the pest’s invasion into the north. If *P. semipunctata* has the opportunity to arrive in Shandong Province further north, the climatic limitations this pest is going to span would be very different than before. Additionally, considering that the *P. semipunctata* is native to the island, we believe that paths of transmission from the islands to mainland China may be more in line with the pest’s colonization routes. Therefore, it is important to emphasize the spread from Hainan Island and Taiwan Island to the mainland. However, Tibet, with plateau alpine climates, could prevent the further distribution of *P. semipunctata* according to the current environmental variable restrictions. It is reported that *P. semipunctata* has already been intercepted in Wuhan City, Hubei province [47]. If *P. semipunctata* were to colonize the interior provinces, the climatic limitations that *P. semipunctata* breaks through would further increase the risk of its spread. Therefore, anthropogenic proliferation needs to be taken more seriously than before. These predictions of the spread direction are based on the invasion of *P. semipunctata* in southern China. Therefore, if the climate warming scenarios proposed in this study are accurate, all the projections could happen in the future.

Some limitations exist in our modeling. First, despite our predictions showing the possibility of alterations in the potential distribution of *P. semipunctata*, we did not provide absolute predictions. The suitable distributions under future climate scenarios are predicted according to the assumption that there is no spread limitation for *P. semipunctata* into the new habitats. Therefore, our projection is based on an ideal state. In other words, the current climate ecological niche of the pest is relatively conservative. Hence, the projected alterations can only serve as a reference for future surveillance for *P. semipunctata* and need to be verified by field investigations and long-term monitoring. Second, although the precipitation of the coldest quarter was selected to be a relatively important variable in the modeling of *P. semipunctata*. The correlation between precipitation and habitat suitability should be interpreted with caution. The complex relationships between hydrothermal conditions and physiological processes affect the lifecycle of *P. semipunctata*. Research has found that the developmental biology in California shows a different pattern from what was observed in Israel [10]. In some regions, a small proportion of the population took two years to emerge as adults. However, these individuals with prolonged development can emerge during a narrow window of time between early June and early July. Therefore, these findings showed that precipitation is not a sufficient condition for the emergence of adults. Finally, our study used relatively limited occurrence data and 23 environmental variables for modeling the pest. Although the RF model exhibited excellent predictive performance, the small sample size and few environmental variables may still affect the accuracy of the model results. In summary, the conservatism of the prediction range appeared to be more prominent. We did not consider the effects of other factors, such as species self-diffusion ability, species interactions, vegetation types, soil type, geomorphological characteristics, and human activity, which may be an important reason for the gaps between the realistic distribution of *P. semipunctata* and the predicted distribution [29]. Therefore, continuous monitoring and incorporating more environmental factor modeling are bound to better address these gaps in the future.

*P. semipunctata* is the worst forestry pest worldwide, where it is transmitted through trade transportation via adults, larvae, pupae, or eggs hidden in wood and wood packaging materials [8]. The main cause of its rapid spread throughout the world has been the transport of Eucalyptus wood. In addition, the exceptional flying capacity and survival of the adult *P. semipunctata* are the basis of local dispersal. Incoming! *P. semipunctata* that have colonized Guangdong, China, showed many difficulties for forest managers in the coming decades. Despite the scale of the changes in suitable areas varying among the scenarios, better monitoring and management are required in these areas. First, the synthesized field survey should be conducted immediately based on the current distribution conditions of *P. semipunctata* in China. Secondly, different measures should be taken according to the occurrence situation in different regions to control the spread of *P. semipunctata*. The forestry department should cooperate fully with the China Entry-Exit Inspection and Quarantine Bureau and the Environmental Protection Department to formulate preventive and control measures for *P. semipunctata* as soon as possible. We can conduct regular surveys to identify early incidence and susceptible forests based on maps of projected potential distributions. The propagation direction of pest populations should be monitored in real time, which will be beneficial for us to determine when pest-control activity is needed. Finally, robust forest monitoring and reporting systems should be established to ensure timely warnings of the effects of climate change on pests and hosts, and to measure the effectiveness of management responses.

## 5. Conclusions

The RF was used to model the potential distribution of *P. semipunctata* in China under future climate scenarios. The results showed that temperature seasonality and the precipitation of the coldest quarter are key environmental factors limiting the current distribution of *P. semipunctata*. Currently, *P. semipunctata* colonizes the triangle zone composed of Zhanjiang City, Qingyuan City, and Jieyang City, Guangdong Province, with the projected potential suitable distribution area of 50.88 × 10^4^ km^2^. Under the future climate scenario, the total suitable distribution area is projected to increase, mainly from Fujian Province toward the north and Guangxi Province toward the north. Based on these findings, we inferred the possible propagation direction of *P. semipunctata* in the future. Furthermore, the prioritized measures for pest control strategies were formulated. However, ignoring some environmental factors resulted in our prediction results being somewhat conservative. Therefore, continuous monitoring and incorporating more environmental factor modeling are bound to better address the gaps between the realistic distribution and the predicted distribution in the future.

## Figures and Tables

**Table 1:** Description of environmental variables used for modeling.

Data Source	Category	Environmental Variables (Unit)	Abbreviation
WorldClim	Bioclimatic	Annual mean temperature (°C)	Bio1
		Mean diurnal range (°C)	Bio2
		Isothermality (%)	Bio3
		Temperature seasonality(°C)	Bio4
		Maximum temperature of warmest month (°C)	Bio5
		Minimum temperature of coldest month (°C)	Bio6
		Temperature annual range (°C)	Bio7
		Mean temperature of wettest quarter (°C)	Bio8
		Mean temperature of driest quarter (°C)	Bio9
		Mean temperature of warmest quarter (°C)	Bio10
		Mean temperature of coldest quarter (°C)	Bio11
		Annual precipitation (mm)	Bio12
		Precipitation of wettest month (mm)	Bio13
		Precipitation of driest month (mm)	Bio14
		Precipitation seasonality	Bio15
		Precipitation of wettest quarter (mm)	Bio16
		Precipitation of driest quarter (mm)	Bio17
		Precipitation of warmest quarter (mm)	Bio18
		Precipitation of coldest quarter (mm)	Bio19
Geospatial data cloud	Terrain	Altitude (m)	Alt.
		Aspect (degree)	Asp.
		Slope (degree)	Slop.

**Table 2:** Evaluation indicators for model accuracy.

Index	Extremely High	Very High	High	Average	Fail
AUC	1.0–0.9	0.9–0.8	0.8–0.7	0.7–0.6	0.6–0.5
Kappa	1.0–0.85	0.85–0.74	0.74–0.65	0.65–0.5	<0.5
TSS	1.0–0.81	0.81–0.74	0.74–0.61	0.61–0.5	<0.5

**Table 3:** Bioclimatic profile of *P. semipunctata* in China.

Environmental Variables	Min.	Max.	Mean	SD	5%	10%	50%	90%	95%
Annual mean temperature (°C)	18.9	23.8	22.1	0.9	18.9	19.1	22.3	23.8	23.8
Mean diurnal range (°C)	7.1	7.6	7.3	0.1	7.1	7.1	7.3	7.5	7.5
Isothermality (%)	25.1	25.8	25.4	0.3	25.1	25.1	25.3	25.7	25.8
Temperature seasonality(°C)	755.7	805.4	765.5	8.9	755.7	755.7	761.8	775.3	775.9
Maximum temperature of warmest month (°C)	31.9	35.1	32.8	1.1	31.9	32.1	32.7	34.3	35.1
Minimum temperature of coldest month (°C)	−6.5	8.2	6.7	5.6	−6.5	−3.8	6.2	7.5	8.2
Temperature annual range (°C)	28.4	30	28.8	0.3	28.4	28.4	28.7	29.2	29.3
Mean temperature of wettest quarter (°C)	27.9	29.6	28.5	1	27.9	28.1	28.5	29.1	29.6
Mean temperature of driest quarter (°C)	13.1	17.2	15.4	0.8	13.2	14.1	15.2	16.7	17.2
Mean temperature of warmest quarter (°C)	27.9	29.6	28.5	1	27.9	28.1	28.5	29.1	29.6
Mean temperature of coldest quarter (°C)	13.1	17.2	15.4	0.8	13.2	14.1	15.2	16.7	17.2
Annual precipitation (mm)	912	1010	977.8	24.5	944	946	974.5	1010	1010
Precipitation of wettest month (mm)	165	182	176.2	4.4	170	170	175.5	182	182
Precipitation of driest month (mm)	8	10	9.4	0.5	9	9	9	10	10
Precipitation seasonality	76.9	78.2	77.4	0.4	76.9	76.9	77.3	77.8	78
Precipitation of wettest quarter (mm)	475	519	504.9	10.6	490	491	503.5	519	519
Precipitation of driest quarter (mm)	29	35	33	1.7	31	31	32.5	35	35
Precipitation of warmest quarter (mm)	415	469	452.5	12.2	436	437	451	469	469
Precipitation of coldest quarter (mm)	29	35	33	1.7	31	31	32.5	35	35

## Data Availability

The raw data supporting the conclusions of this article will be made available by the authors on request.

## Abbreviations

The following abbreviations are used in this manuscript:

*P. semipunctata*	*Phoracantha semipunctata*
RF	Random Forests
